# Alport Syndrome: From Pathogenesis to a Potential Therapy

**DOI:** 10.1371/journal.pmed.0030154

**Published:** 2006-03-07

In 1927, Cecil Alport described a family in which affected individuals developed progressive kidney failure, deafness, and sometimes eye problems. Alport syndrome, although it affects only one in 50,000 live births in the United States, is the second most commonly inherited reason for kidney failure. It is caused by mutations in the genes that encode type IV collagen, a structural component of the thin, sheet-like basement membrane that covers the glomeruli, the kidney's filtration units. The glomerular basement membrane (GBM) normally filters fluid and small molecules (but not proteins or red blood cells) from the capillaries in the glomeruli into the urine, but in Alport syndrome, the collagen scaffold of the GBM is defective and, over time, the GBM splits and thins. The first symptom of Alport syndrome is blood in the urine (hematuria), followed by proteinuria and progressive renal failure as scar tissue (fibrotic tissue) forms around the glomeruli. The syndrome has no specific treatment, but kidney transplantation is usually successful in patients with end-stage kidney failure.

There are six isoforms of type IV collagen—α1(IV) through α6(IV). In immature kidneys, the GBM contains α1(IV) and α2(IV), but these are replaced by α3(IV), α4(IV), and α5(IV) as the kidneys mature. The gene encoding α5(IV) is mutated in patients with X-linked Alport syndrome (85% of cases); mutations in the genes encoding α3(IV) or α4(IV) cause other forms of the syndrome. In all cases, the normal switch in collagen isoforms does not occur as the kidneys mature. Raghu Kalluri and colleagues have been investigating whether this failure to switch might make the GBM more susceptible to proteolytic degradation by matrix metalloproteinases (MMPs). They now report that MMPs have a dual role during disease progression in a mouse model of Alport disease, and suggest that MMP inhibition might be therapeutic during the early stages of the human disorder.

The mouse model used by Raghu Kalluri, Michael Zeisberg, and colleagues to test their ideas about the pathogenesis of Alport syndrome is the α3(IV) ^−/−^ mouse, which lacks functional α3(IV) collagen. These mice are born normal, but by four to five weeks old, their GBMs begin to disintegrate and they develop proteinuria. By eight weeks old, fibrotic tissue has formed in the tubulointerstitial compartment of their kidneys, and the mice die by 14 weeks old from kidney failure. The researchers first examined the localization of MMP-2, MMP-3, and MMP-9 (all of which degrade GBM) in α3(IV) ^−/−^ mice. Wild-type mice expressed low levels of these MMPs in their kidneys, but α3(IV) ^−/−^ mice expressed increased levels of MMP-2 and MMP-3 in their glomeruli at four weeks old, and as their disease progressed, expression of all three MMPs spread to the renal tubulointerstitial compartment. Renal expression of these MMPs was also increased in patients with X-linked Alport syndrome and end-stage renal failure when compared with normal kidneys. Furthermore, GBM from humans with Alport syndrome and from α3(IV) ^−/−^ mice was more susceptible to MMP degradation than that from normal humans or mice.

These results support the idea that increased proteolytic degradation of a defective GBM may be responsible for Alport syndrome, but renal disease still occurs in α3(IV) ^−/−^ mice when MMP-9 is missing. Could other MMPs compensate for the loss of MMP-9? When the researchers examined renal tissue from α3(IV) ^−/−^ mice deficient for MMP-2 and/or MMP-9, they did indeed discover compensatory upregulation of other MMPs. So the researchers then looked to see whether pharmacological agents that inhibit multiple GBM-degrading MMPs could alter disease progression in α3(IV) ^−/−^ mice. The researchers report that giving such drugs to four-week-old mice (before proteinuria developed) delayed disease progression and increased their survival by five weeks. However, giving the same drugs to eight-week-old mice (in which there was tubulointerstitial fibrosis) shortened their lives by two to three weeks.

Based on these animal experiments, the researchers suggest that in patients with Alport syndrome, the GBM (unlike GBM in healthy individuals) is susceptible to degradation by the low levels of MMP normally present in the kidney. Partial disruption of the GBM attracts infiltrating monocytes, which increase local MMP concentrations and accelerate GBM destruction. MMP inhibitors during this phase of the syndrome should be protective, suggest the researchers, but once the damage is sufficient to stimulate renal fibrosis, the same drugs will accelerate disease progression by inhibiting the MMPs that normally help to degrade fibrotic tissue. MMP inhibitors are already being developed for other indications and would be worth investigating as preventive drugs in Alport syndrome. But, warn Kalluri and colleagues, these drugs would be a double-edged sword, and could only be used in patients with identified genetic defects and only before the onset of proteinuria.

## 

**Figure:**
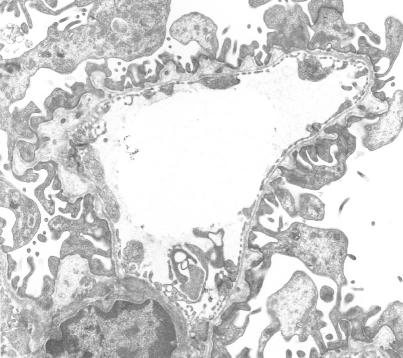
Transmission electron microscopy of a kidney from an α3(IV) ^−/−^ mouse. Without treatment, glomerular basement membrane lesions and podocyte effacement cause severe proteinuria in these mice.

